# Vitronectin-based hydrogels recapitulate neuroblastoma growth conditions

**DOI:** 10.3389/fcell.2022.988699

**Published:** 2022-10-11

**Authors:** Ezequiel Monferrer, Oana Dobre, Sara Trujillo, Mariana Azevedo González Oliva, Alexandre Trubert-Paneli, Delia Acevedo-León, Rosa Noguera, Manuel Salmeron-Sanchez

**Affiliations:** ^1^ Department of Pathology Medical School, University of Valencia-INCLIVA Biomedical Health Research Institute, Valencia, Spain; ^2^ Low Prevalence Tumors, Centro de Investigación Biomédica En Red de Cáncer (CIBERONC), Instituto de Salud Carlos III, Madrid, Spain; ^3^ Centre for the Cellular Microenvironment, Advanced Research Centre, University of Glasgow, Glasgow, United Kingdom; ^4^ INM—Leibniz Institute for New Materials, Saarbrücken, Germany; ^5^ Clinical Analysis Service, Hospital Universitario Dr. Peset, Valencia, Spain

**Keywords:** vitronectin, neuroblastoma, polyethylene-glycol, stiffness, extracellular matrix, digital image analysis

## Abstract

The tumor microenvironment plays an important role in cancer development and the use of 3D *in vitro* systems that decouple different elements of this microenvironment is critical for the study of cancer progression. In neuroblastoma (NB), vitronectin (VN), an extracellular matrix protein, has been linked to poor prognosis and appears as a promising therapeutic target. Here, we developed hydrogels that incorporate VN into 3D polyethylene glycol (PEG) hydrogel networks to recapitulate the native NB microenvironment. The stiffness of the VN/PEG hydrogels was modulated to be comparable to the *in vivo* values reported for NB tissue samples. We used SK-N-BE (2) NB cells to demonstrate that PEGylated VN promotes cell adhesion as the native protein does. Furthermore, the PEGylation of VN allows its crosslinking into the hydrogel network, providing VN retention within the hydrogels that support viable cells in 3D. Confocal imaging and ELISA assays indicate that cells secrete VN also in the hydrogels and continue to reorganize their 3D environment. Overall, the 3D VN-based PEG hydrogels recapitulate the complexity of the native tumor extracellular matrix, showing that VN-cell interaction plays a key role in NB aggressiveness, and that VN could potentially be targeted in preclinical drug studies performed on the presented hydrogels.

## Introduction

The extracellular matrix (ECM) is a three-dimensional (3D) network that provides support and structure to tissues. The interaction between ECM, cells, tissue vascularization, lymphatic vessels, and nerve fibers is essential for tissue biotensegrity (transmission of mechanical forces and their stability). ECM alterations in structure, composition, stiffness or organization result in tissue dysfunction such as cell denervation, loss of regeneration potential, aberrant wound-healing capacity, and inflammation (Estofolete et al., 2010; Lu et al., 2011; Tomlin and Piccinini, 2018). Moreover, the ECM contributes to the biotensegrity of the tumor, leading to genetic and epigenetic modifications (Noguera et al., 2012; Tadeo et al., 2014). Hence, current oncology research focuses on the role of the ECM in cancer aggressiveness, progression, and therapy resistance (Jinka et al., 2012; Walker, Mojares and Del Río Hernández, 2018). The ECM glycoprotein vitronectin (VN) is an adhesive molecule that presents multiple binding domains; this allows VN to interact with different ECM molecules and integrins, which have been shown to promote tumor cell migration (Felding-Habermann et al., 2001; Shi et al., 2015; Schneider et al., 2016). Therefore, the inhibition of VN interactions with tumor cell integrins and/or its ECM remodeling action could impede cell migration and tumor spreading and provide an effective therapeutic approach. Previous studies on human neuroblastoma (NB) biopsies, one of the most common solid cancers in childhood (Maris et al., 2007; Kaatsch, 2010), have shown that ECM morphological patterns related to collagen fibers, glycosaminoglycans, and glycoproteins, as well as structural characteristics of their blood vascularization and lymph vessels, can define high-risk and even ultra-high-risk NB aggressiveness (Tadeo et al., 2016, 2017, 2018). In particular, our previous results highlighted VN as a relevant glycoprotein related to NB patients with poor prognosis (Burgos-Panadero, Noguera, et al., 2019; Vicente-Munuera et al., 2020). Our previous results with a clinical cohort and preclinical models (orthotopic xenograft VN knock-out (KO) mice and 3D bioprinted hydrogels with different stiffness) have also established that the interaction of VN, its ligands (e.g., αv integrins), and genomic intratumor heterogeneity in *MYCN*-amplified NB cell line are related to increased ECM stiffness (Burgos-Panadero, Noguera, et al., 2019; López-Carrasco et al., 2020; Monferrer, Martín-Vañó, et al., 2020; Monferrer, Sanegre, et al., 2020; Vicente-Munuera et al., 2020). Furthermore, we performed preclinical therapeutic studies on NB monolayer cell cultures, targeting VN function blockade by employing cilengitide (αv integrin antagonist) and combination therapy with etoposide-loaded (cytotoxin used in high-risk NB treatment) lipid nanoparticles (Burgos-Panadero et al., 2021). Despite our previous observations suggesting a high synergy between cilengitide and etoposide, the system did not recapitulate the VN expression pattern nor the 3D cell growth observed in NB tumors. Considering all the presented arguments and the inefficiency of current models, a more reliable and physiologically relevant 3D NB *in vitro* model is needed.

*In vivo* models such as VN-KO NB mice xenografts have demonstrated that neuroblasts synthesize VN analogously to human NB tumors when growing in 3D conditions surrounded by the tumorous ECM (Burgos-Panadero, Noguera, et al., 2019) however, these *in vivo* models are challenging to produce, reproduce, and analyze. 3D *in vitro* models are versatile platforms that can be designed to closely recapitulate the cancer pathophysiology and study ECM-dependent tumor behavior (Kenny and Bissell, 2003; Denys et al., 2009; Lejeune and Álvaro, 2009; Imamura et al., 2015; Doyle and Yamada, 2016; Gao et al., 2017; Kuen et al., 2017); however, most models incorporate bioactive elements, such as gelatin, that can compete with the protein of interest and yield debased results. In contrast, polyethylene glycol (PEG) is a biocompatible and bioinert polymer that enables hydrogel formation by covalent biomolecule incorporation. Protein-based hydrogels can be designed with specific composition, properties, and biological functionality, allowing the study of the role of various biomolecules (Trujillo et al., 2020; Dobre et al., 2021). Here, we incorporated full-length VN in PEG hydrogels (VN/PEG) to create a tunable 3D cell culture platform that can recapitulate the NB microenvironment and simulate high-risk NB behavior.

In this work, we firstly characterized the bioactivity of the VN/PEG gels, their mechanical properties, and the viability of encapsulated SK-N-BE (2) cells. We subsequently investigated the relation between PEGylated and cellular VN to assess the behavior of SK-N-BE (2) in the system. Our results demonstrate the suitability of the engineered VN/PEG models to mimic NB behavior and their aptitude to be used as drug testing platforms.

## Materials and methods

### Vitronectin PEGylation

**FIGURE 1:**
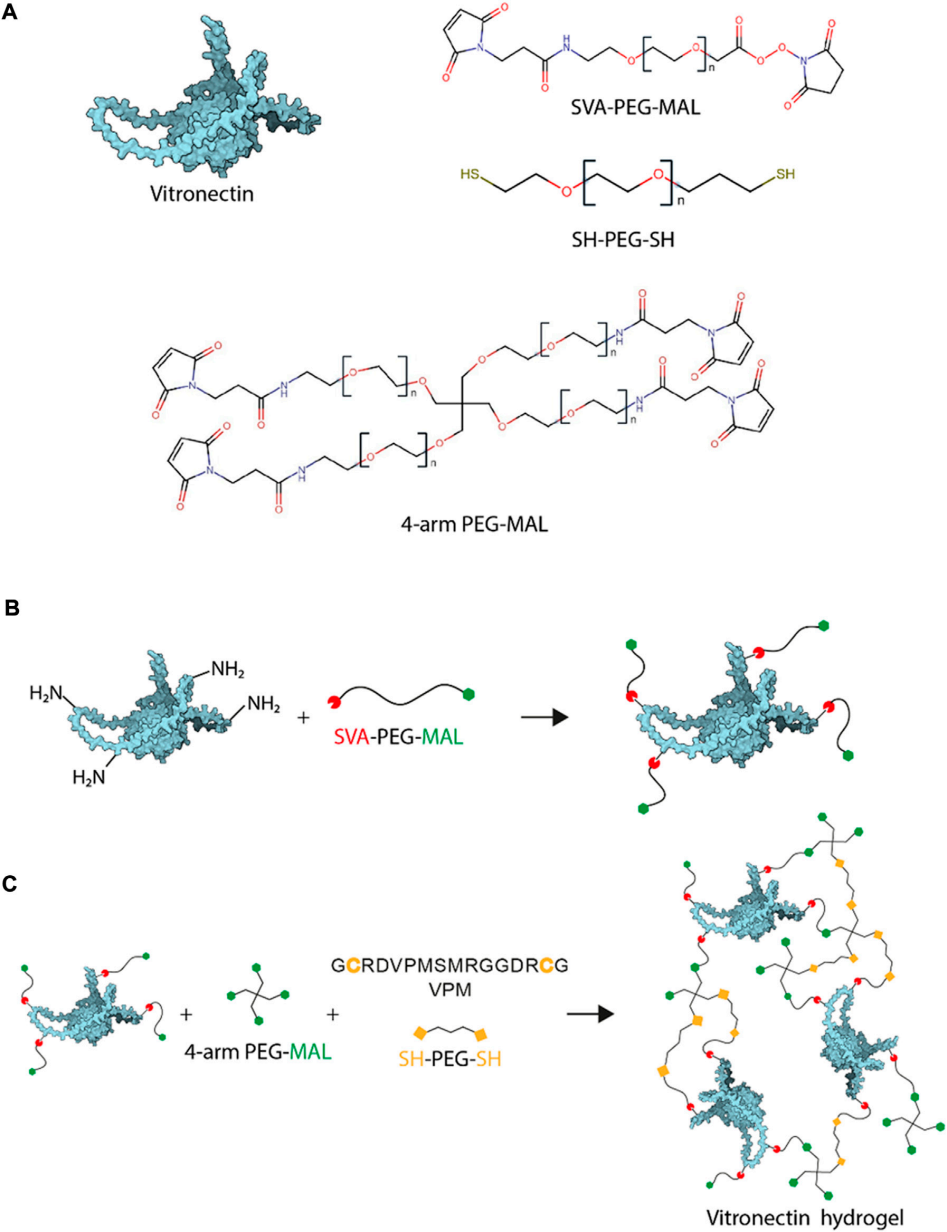
VN-based hydrogel chemistry. **(A)**. Gel components: vitronectin structure, SVA-PEG-MAL, SH-PEG-SH and 4-arm PEG-MAL. **(B)**. VN PEGylation is the reaction used to functionalise VN molecules with MALs at different ratios and **(C)**. VN/PEG gel formulation schematic drawing prepare using Michal type addition, reaction used to crosslink the MAL to SH.

Vitronectin (VN, Peprotech, 1 mg) was PEGylated by modifying a previously published procedure (Almany and Seliktar, 2005). VN was dissolved in NaHCO_3_ 0.1 M (pH 8.5). Subsequently, Maleimide-PEG-Succinimidyl Valerate (MAL-PEG-SVA, 3.4 kDa, LaysanBio) was mixed at a mass ratio VN:SVA 1:4 and incubated for 1 h at room temperature (RT) (Figure 1). The product of the reaction was dialyzed (Mini-A-Lyzer, MWCO 10 kDa, ThermoFisher) against PBS for 1 h at RT and stored at −20°C or immediately used. The degree of PEGylation for different VN:SVA ratios was measured by tracking the reduction in primary amines after the reaction using a 2,4,6-trinitrobenzene sulfonic acid (TNBSA, ThermoFisher) assay, which indicated an increase in the VN degree of modification as the VN:SVA ratio increased (Supplementary Figure S1).

### PEGylated VN bioactivity

**FIGURE 2:**
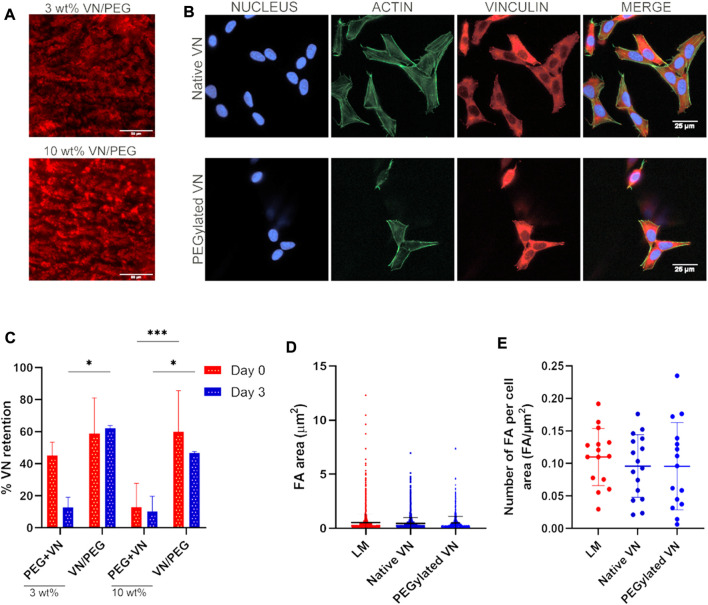
VN distribution and bioactivity after incorporation in the 3D hydrogel. **(A)**. Immunofluorescence images of VN distribution in 3 and 10 wt% VN/PEG gel cryosections (scale bar 50 µm). VN was stained with a Cy3 secondary antibody, shown in red in the images. **(B)**. Representative fluorescence images (nucleus in blue, actin in green and vinculin in red) of SK-N-BE (2) cells seeded on top of unmodified or PEGylated VN for 24 h (scale bar: 25 µm). Insets of FA images are shown in Supplementary Figure S3. **(C)**. Immunofluorescence images of VN distribution in 3 and 10 wt% VN/PEG gel cryosections were quantified by QuPath 0.3.2 to calculate VN retention after 3 days. **(D)**. The area of the focal adhesions was found comparable between all conditions. **(E)**. The same trend was observed after analysis focal adhesion density. For each experiment, 15 images (3 replicates, five images each) were analyzed per sample condition.

Cell adhesion formation analysis in SK-N-BE (2) cells was performed to assess VN bioactivity compared to that of laminin (positive control) and ensure maintenance of VN bioactivity after PEGylation. First, protein solutions at 20 μg ml^−1^ were added on top of a sterile coverslip for 1 h at RT. Subsequently, 2 × 10^4^ SK-N-BE (2) cells were seeded onto each protein coated coverslip substrate and cultured in growth media (Iscove’s Modified Dulbecco’s Media (IMDM)) without fetal bovine serum (FBS) to avoid unspecific focal adhesion formation. After 24 h, cells were fixed, permeabilized, and blocked for antibody incubation; primary mouse monoclonal-anti-vinculin antibody (Sigma, 1:400) was added and incubated for 1 h at RT, samples were washed thrice in PBST (0.1% Tween 20 (Sigma)) before incubation of secondary Cy3 rabbit-anti-mouse antibody (Jackson ImmunoResearch, 1:200) and Alexa Fluor 488 Phalloidin 1:400, ThermoFisher) for 1 h at RT. Finally, samples were washed thrice in PBST and mounted with VECTASHIELD/DAPI mounting media (Vector Laboratories). Images were taken by a Zeiss Axio Observer. Z1 at ×63 magnification (shown in Figure 2) and later assessed by CellProfiler 4.2.1 to measure focal adhesions size and density. We used a custom pipeline with different steps that include the identification of objects. For focal adhesion calculations, we set up a threshold for vinculin-stained images using an identified object function and calculate the focal adhesion area. At least five images per replicate were taken in three independent experiments.

### Hydrogel formation

PEG hydrogels were formed using Michael-type addition reaction under physiological pH and temperature following a previously published protocol (Phelps et al., 2010). Briefly, a final concentration of 500 μg ml^−1^ of PEGylated VN was added to different amounts of 4-arm-PEG-MAL (PEG-MAL, 3 wt% or 10 wt%). The thiolated crosslinker was always added at the end, at a ratio 1:1 maleimide:thiol to ensure full crosslinking. The crosslinker used was a 9:1 mixture of PEG-dithiol (SH-PEG-SH, 2 kDa, LaysanBio) and protease-degradable peptide, flanked by two cysteine residues (VPM peptide, GCRDVPMSMRGGDRCG, purity 96.9%, Mw 1,696.96 Da, GenScript) (Figure 1). For cell seeding, the cell suspension was mixed with the VN and PEG-MAL before addition of the crosslinker. Once the crosslinker was added, samples were incubated for 30 min at 37°C to allow gelation. PEG only hydrogels were produced as negative controls. Some PEG hydrogels incorporated native VN (non-PEGylated) instead of PEGylated VN. The nomenclature used in this manuscript is x wt% PEG for hydrogels without VN, x wt% VN/PEG for hydrogels with PEGylated VN, and × wt% PEG + VN for hydrogels with native VN, x being the percentage of PEG-MAL used.

### Vitronectin release

VN release at 72 h was measured to demonstrate that PEGylated VN was covalently crosslinked to the PEG network; to assess this, plain hydrogels (3 and 10 wt% VN/PEG, PEG + VN or PEG only) were immersed for 72 h in PBS. Native VN was incorporated as previously indicated for PEGylated VN. Subsequently, hydrogels were embedded in optimal cutting temperature compound (OCT compound, VWR) and flash frozen in liquid nitrogen to preserve gel structure. Samples were stored at −80°C until use. A cryostat (Leica, −20°C) was used to cut the samples in 50 μm thick sections and presented on a microscope slide for immunostaining (Superfrost™ Plus, ThermoFisher). Five images were taken per replicate in triplicated samples, with the same exposure time, by a ZEISS AxioObserver Z.1 at ×40 magnification and then quantified by QuPath 0.3.2 to calculate the VN area in relation to the total image area (%).

The media of each condition were systematically collected across 14 days of culture, concentrated with SpeedVac Vacuum Concentrator (ThermoFisher) until volumes were equivalent, and quantified by ELISA colorimetric assay for VN (R&D Systems) to assess the amount of cellular VN secreted in that culture period (Table 1). The optical density of each well was determined by a micro-plate reader set to 450 nm. Samples were measured in duplicate.

**TABLE 1:** Summary of ELISA assay for secreted VN detection in the culture media at different time points. VN detected values are referred as ng of VN secreted per hydrogel each day.

	Collection day	Hydrogels (n of samples)	ngVN/HG day
3 wt% PEG	3	12	1.29
	6	8	0.60
	7	8	—
	8	4	—
	10	4	—
	13	4	—
	14	4	—
3 wt% VN/PEG	3	12	0.95
	6	8	0.89
	7	8	—
	8	4	—
	10	4	—
	13	4	—
	14	4	—
10 wt% PEG	3	12	0.49
	6	8	—
	7	8	—
	8	4	—
	10	4	—
	13	4	—
	14	4	—
10 wt% VN/PEG	3	12	0.48
	6	8	—
	7	8	1.16
	8	4	—
	10	4	—
	13	4	—
	14	4	—

### Vitronectin immunostaining

VN was detected *via* immunofluorescence in hydrogel cryosections. Sections were dehydrated with EtOH 100% for 10 min at RT, dried, and rehydrated with two PBS washes of 15 min. The sections were blocked for 30 min and incubated with the primary anti-VN antibody (Invitrogen, 1:200) for 1 h. The samples were washed three time in PBST and then incubated in secondary Cy3 anti-mouse antibody (Jackson ImmunoResearch, 1:200) for 1 h. Samples were washed, mounted, and imaged as previously described.

### Mechanical properties

Nanoindentation measurements were performed using a Chiaro Nanoindenter (Optics 11) mounted on top of an inverted phase contrast microscope (Evos XL Core, Thermofisher), following a previously described approach (Bartolozzi et al., 2020). Measurements were performed at RT in culture media. The area of the sample was mapped defining square areas (2,500 μm^2^, 25 measurements); a minimum of three maps per replicate were measured and at least two replicates per sample were tested. The selected cantilever had a stiffness of 0.46 Nm^−1^ and held a spherical tip of 52 µm radius. The collected curves were analyzed using a custom Python code (Ciccone et al., 2022). Curves were first aligned using a baseline detection method based on the histogram of the force signal (Duanis-Assaf, Razvag and Reches, 2019) and the corresponding indentation was calculated for each curve. The analysis was performed using the Hertz model for a spherical indenter to fit the curves obtained.

### Cell culture

We chose SK-N-BE (2) *MYCN*-amplified human NB cell line to be encapsulated into the hydrogels since its genetic characteristics are representative for 50% of high-risk NB cases (Otte et al., 2021). SK-N-BE (2) were acquired from American Type Culture Collection (ATCC, Manassas, VA, United States) and expanded in IMDM culture (Gibco, Thermofisher), supplemented with 10% FBS (Thermofisher), 1% Insulin-Transferrin-Selenium G Supplement (ITS, Thermofisher) and 1% penicillin/streptomycin (Thermofisher) at 37°C and 5% CO_2_ atmosphere. Cells were seeded at 2 × 10^6^ cells ml^−1^ and kept under culture conditions analogous to the expansion process. Hydrogels were cultured from 1 to 7 days; the media was exchanged every 3 days.

### Live/dead viability assay

Cellular viability was tested by standard a LIVE/DEAD assay (ThermoFisher). Briefly, SK-N-BE (2) cells were encapsulated at 2 × 10^6^ cells ml^−1^ to allow single-cell analysis. At each time point, gels were washed twice with PBS, and calcein-AM (4 µM) and Ethidium Homodymer-1 (2 µM) were added for 15 min at 37°C; subsequently, gels were washed twice and imaged using a ZEISS AxioObserver Z.1. Samples were imaged on day 1, 5 and 7 at ×10 magnification and analyzed by Fiji to project the overall viability (%), cluster size (µm^2^), and cluster density; five images were taken per replicate in triplicated samples.

### Statistical analysis

The statistical analysis was performed using GraphPad Prism 9.1.2 software. All *in vitro* experiments were carried out in triplicate unless stated otherwise. All graphs represent mean ± standard deviation (SD) unless stated otherwise. The goodness of fit of all datasets was assessed *via* D’Agostino-Pearson Normality test or Shapiro-Wilk test. When comparing three or more groups: normal distributed populations were analyzed *via* analysis of variance test (ANOVA test) performing a Tukey’s post hoc test to correct for multiple comparisons; when populations were not normally distributed, a Kruskal–Wallis test was used with a Dunn’s post hoc test to correct for multiple comparisons. When comparing only two groups, parametric (normal distributed population, *t*-test) or nonparametric (Mann-Whitney test) tests were performed. Differences among groups are stated as follows: for *p*-values <0.05 (*), when *p*-values <0.01 (**), for *p*-values < 0.005 (***), for *p*-values < 0.001 (****), when differences between groups are not statistically significant (ns).

## Results

### PEGylation allows fabrication of hydrogels with functional full-length VN

Prior to hydrogel formation, VN was PEGylated *via* a Michael-type addition reaction by selective functionalization of the VN lysine residues with Maleimide-PEG-Succinimidyl Valerate (MAL-PEG-SVA). According to the results obtained from 2,4,6-Trinitrobenzene Sulfonic Acid (TNBS) assay, from the total VN amines, 99.1% ± 1.1, 99.0% ± 1.4 and 96.8% ± 1.8 remained free after VN PEGylation with 1:10, 1:4 and 1:1 SVA:NH_2_ molar ratios, respectively (Supplementary Figure S1). Since the reaction yield was less than 4%, we PEGylated VN with an excess of SVA, at mass ratio VN to MAL-PEG-SVA of 1:4 (approximately 1:60 M ratio) (Supplementary Figure S1) to ensure enough VN PEGylation.

If VN is covalently crosslinked there should not be any VN release, whereas the addition of native VN would cause significant release of VN by diffusion. To demonstrate that VN was crosslinked to the hydrogel network, we assessed the presence of PEGylated VN within the VN/PEG hydrogels after 3 days *via* immunostaining and compared it to the native VN in the PEG + VN hydrogels (Figure 2A, Supplementary Figure S2). Hydrogels without VN did not show any staining (Supplementary Figure S2) and both VN/PEG and PEG + VN hydrogels showed VN detection at day 0, demonstrating that VN was initially loaded in the hydrogels. VN/PEG hydrogels did not release any VN after 3 days (Figure 2C), which was retained in the hydrogel in a concentration similar to the initial one (day 0). However, 3 wt% PEG + VN released approximately 70% of the initial VN after 3 days (Figure 2C). Interestingly, 10 wt% PEG + VN poorly incorporated VN when compared with 10 wt% VN/PEG.

We then demonstrated that PEGylation does not influence the biological activity of VN. We investigated focal adhesion formation of SK-N-BE (2) cells cultured over substrates coated with either laminin, native VN, or PEGylated VN (Figure 2B and Supplementary Figure S3). We observed comparable cell spreading area on all substrates. We have monitored SK-N-BE (2) cell attachment over time. We observed that culturing cells for 24 h will allow better cell adhesion compared to 4 h in culture. Also, we did not observe any differences in either size or number of focal adhesions between laminin, native and PEGylated VN (Figures 2D,E). These experiments demonstrate that cells interact with PEGylated VN using cell-adhesion domains that were not blocked by PEG.

### VN does not modify initial hydrogel stiffness

**FIGURE 3:**
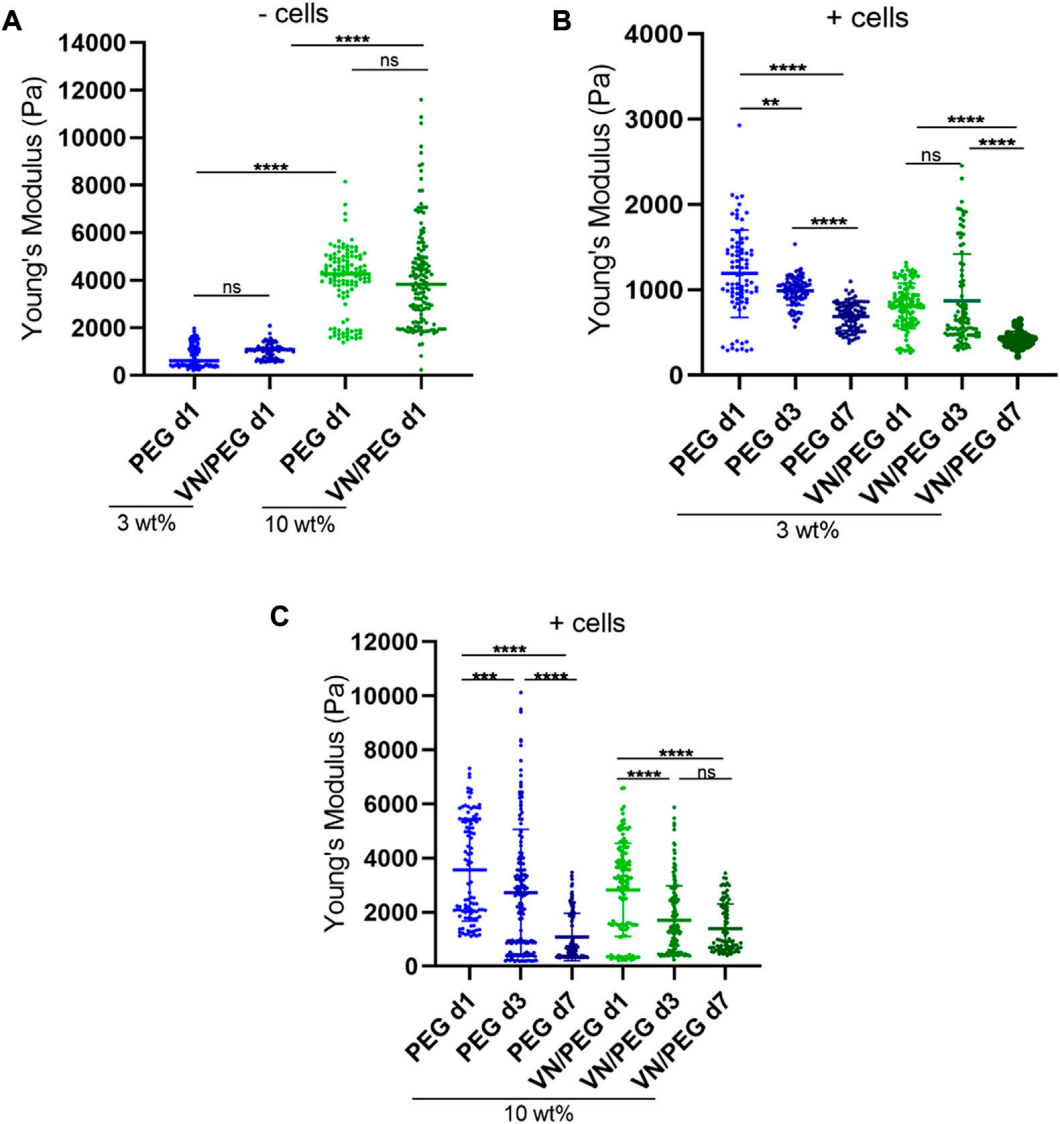
3D VN hydrogel with tunable stiffness. **(A)**. Comparison between 3 and 10 wt% PEG gel stiffness with/without VN measured using nanoindentation. **(B,C)**. *In situ* stiffness measurements of 3 **(B)** and 10 wt% **(C)** SK-N-BE (2)-loaded hydrogels at 1, 3, and 7 days. A minimum of three maps per replicate were measured and at least two replicates per sample were tested. ANOVA test was used for statistical analyzes.

Using PEG as a hydrogel network allows the physicochemical properties of the system to be controlled (Lutolf and Hubbell, 2003; Cambria et al., 2015; Goldshmid and Seliktar, 2017), (Figure 3). Increasing the amount of PEG in the system (from 3 to 10 wt%) increases the Young’s modulus independently of the incorporation of VN in the hydrogel (Figure 3A) as shown for similar systems (Trujillo et al., 2020; Dobre et al., 2021). The 3 wt% PEG hydrogels had a Young’s modulus of 0.81 ± 0.48 kPa and slightly increased to 1.03 ± 0.31 kPa upon incorporation of VN (3 wt% VN/PEG). 10 wt% PEG hydrogels showed an average Young’s modulus of 3.99 ± 1.37 kPa; and 10 wt% VN/PEG displayed 4.12 ± 2.14 kPa. Importantly, these results are similar to NB stiffness, from 0.17 to 8.45 kPa (Bao et al., 2022), and show that VN incorporation into the hydrogels does not significantly modify hydrogel stiffness.

The degradability of the hydrogels was tuned by combining a protease degradable crosslinker (VPM peptide) with SH-PEG-SH in the synthesis of the hydrogels. Using 10 wt% cell-degradable VPM (Figure 1C), the mechanical properties of the hydrogels decreased after 7 days of culture (Figures 3B,C), 42% in 3 wt% PEG; 48% in 3 wt% VN/PEG; 69% in 10 wt% PEG and 68% in 10 wt% VN/PEG. The incorporation of VN in the hydrogels leads to an additional stiffness reduction when cells are encapsulated for both 3 and 10 wt% VN/PEG hydrogels. This is likely due to the degradation of VN covalently linked to the PEG network by the cells, which further contributes to reduce the crosslinking density.

### VN/PEG hydrogels support NB growth

VN incorporation in PEG hydrogels recapitulates part of the high-risk NB ECM, which influences cancer cell behavior, together with the stiffness of the microenvironment. As a proof of concept, VN concentration was kept constant at 500 μg ml^−1^ in the VN/PEG hydrogels, and the stiffness was then modified.

**FIGURE 4:**
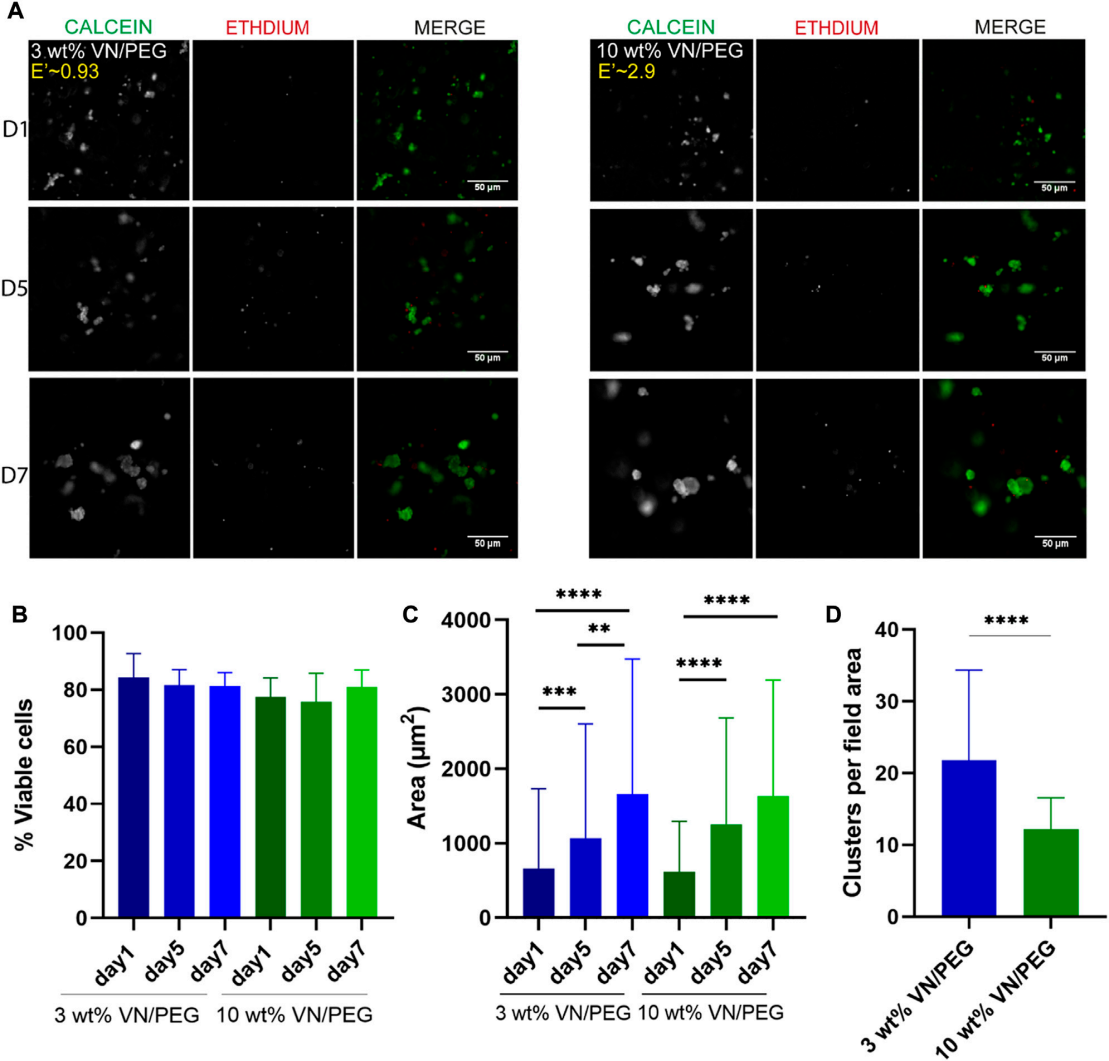
Cell viability in 3D VN based hydrogels. **(A)**. Images of calcein-AM (green) and ethidium homodimer-1 (red) for SK-N-BE (2) in 3 and 10 wt% VN/PEG with 500 μg ml^−1^ of VN. **(B)**. Quantification of SK-N-BE (2) viability with ANOVA statistical method. **(C)**. Cell cluster area for 3 and 10 wt% VN/PEG hydrogels after 1, 5, and 7 days (scale bar 50 µm). Statistical differences were analyzed by Kruskal–Wallis test. **(D)**. Mean number of clusters per field area for 3 and 10 wt% VN/PEG hydrogels, grouping images from 1, 5 and 7 days together. Mann-Whitney analysis was performed to compare the number of clusters. A total of 15 images (3 replicates, 5 images each) were taken per sample condition.

Cell viability assays performed at 1, 5 and 7 days (Figure 4) revealed that approximately 80% of the encapsulated cells were viable after 7 days of culture regardless of the stiffness of the hydrogel (Figures 4A,B). Cell cluster formation was also observed, the size of which increased 2.5-fold from day 1 to day 7 in both 3 and 10 wt% VN/PEG (Figure 4C). Comparing stiffness conditions for each time point revealed no differences in cell cluster size, but the average cluster density was significantly lower in 10 wt% VN/PEG hydrogels (Figures 4C,D).

### SK-N-BE (2) cells synthesize VN in the 3D VN-based hydrogels

Since PEGylated VN is retained into VN/PEG hydrogels but not the native one, we checked whether VN synthesized by neuroblastic cells diffused out of the hydrogels into the culture media. Alternatively, cell-secreted VN might interact with other ECM proteins secreted by the cells within the hydrogel and still be retained in the 3D system contributing to the NB microenvironment: to evaluate this, a colorimetric ELISA assay for cell-secreted VN was performed. VN was mainly detected only when analyzing media from at least eight hydrogels cultured over 3 days (Table 1). Each hydrogel released from 0.48 to 1.29 ng VN day^−1^, with 3 wt% PEG hydrogels showcasing the maximum release rate during the initial culture period. However, no statistical differences could be inferred between conditions (Table 1).

**FIGURE 5:**
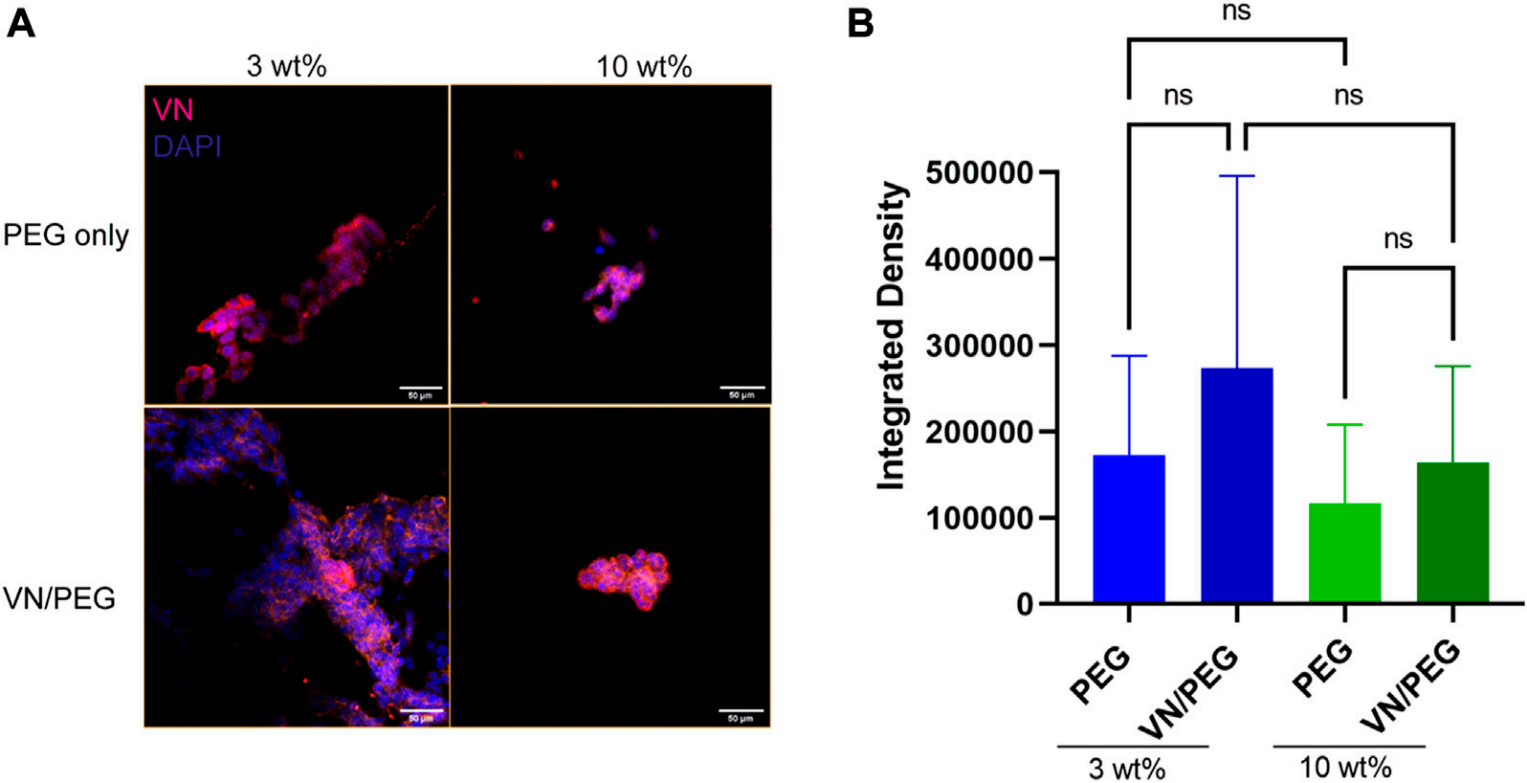
Distribution of VN secreted by the cells in 3D PEG and VN/PEG hydrogels. Immunofluorescence images of SK-N-BE (2) cultured for 7 days in 3 and 10 wt% PEG and VN/PEG. Images show nuclei (blue) and VN (magenta) (scale bar 50 µm). **(B)**. Immunofluorescence quantification of VN secreted by cells in 3 and 10 wt% PEG with/without VN. We have represented in the graph the Integrated density (total raw intensity per image normalized by cell area). A total of 15 images (3 replicates, 5 images each) were taken per sample condition.

Then, the SK-N-BE (2) cells were incorporated in the 3 and 10 wt% PEG hydrogels with/without VN over 7 days and the VN was quantified using immunofluorescence (Figure 5). Confocal images confirmed SK-N-BE (2) VN synthesis, being slightly higher but not statistically significant in 3 than in 10% PEG hydrogels. Interestingly, the same tendency was observed when PEGylated VN was incorporated into the hydrogel. Despite no significant differences being found, VN/PEG hydrogels display higher VN integrated density than PEG hydrogels (Figures 5A,B), suggesting that cells synthesize more VN in VN/PEG hydrogels or that the integrated VN density of VN/PEG hydrogels is the sum of the fluorescence signals from cell synthesized VN together with PEGylated VN.

## Discussion

The physics of cancer is an emerging field of oncology which highlights the mechanical properties of the tumor microenvironment (TME), focusing on the role of cell-ECM interactions in cancer disease and metastasis (Burgos-Panadero, Lucantoni, et al., 2019; Grant et al., 2022; Kpeglo et al., 2022; Liu et al., 2022; Xiao et al., 2022). As glycoproteins interact with integrins and are involved in cell adhesion and migration pathways, molecules similar to VN, such as FN have been used in cancer mechanotransduction studies (Missirlis and Spatz, 2014; Elosegui-Artola et al., 2016). In fact, VN has been shown to play a key role in NB aggressiveness (Burgos-Panadero, Noguera, et al., 2019; Vicente-Munuera et al., 2020), as such, we have developed a new platform based on PEG hydrogels that incorporate full-length VN and allows fully controllable mechanical properties to recreate high-risk NB behavior *in vitro* and to perform future patient-specific preclinical drug testing. Importantly, these models allow cell-cell and cell-ECM crosstalk that cannot be recreated by cell monolayer cultures (Burgos-Panadero et al., 2021), using a less demanding technology when compared with our previous bioprinted gelatin-based models (Monferrer, Martín-Vañó, et al., 2020; Monferrer, Sanegre, et al., 2020). Furthermore, PEG-based hydrogels allow for fine tuning properties for mechanical regulation, which, unlike gelatin models, can be decoupled from degradability and cell adhesion cues. In this way, PEG offers a bioinert synthetic platform that avoids confounding effects of the scaffold that may alter cell behavior, especially when performing drug targeted studies such as cilengitide to block cell-VN interaction. Hence, PEG allows system functionalization by adhesive peptide immobilization, full-length protein addition or growth factor incorporation; this enables step-by-step studies on artificial scaffolds with increasing complexity by independently adding bioactive components, such as collagen, that precisely recreate the TME, thus progressively evolving the model to a more relevant NB physiopathology. Maleimide groups were chosen as moieties to crosslink the hydrogels due to their higher affinity towards thiol groups as well as their shorter gelation times at physiological pH relative to hydrogels fabricated using acrylate groups (Phelps et al., 2012). Moreover, Michael-type addition has been previously used to form bioactive and biocompatible hydrogels (Phelps et al., 2015; Jansen et al., 2018).

Full-length proteins retain high biological activity after the PEGylation process when incorporated into hydrogel systems (Almany and Seliktar, 2005; Seidlits et al., 2011; Francisco et al., 2014); however, no studies on VN PEGylation have been previously reported, consequently, we adapted a FN PEGylation protocol for targeting VN lysine residues (Trujillo et al., 2020). Despite the TNBS assay indicating low PEGylation efficiency (Supplementary Figure S1), data from hydrogel cryosections demonstrated that VN reaction with molar excess of MAL-PEG-SVA increases VN PEGylation efficiency, hence, full-length VN can be covalently incorporated into the PEG system (Supplementary Figure S2, Figures 2A–C). Full-length proteins have been incorporated at maximum final concentration of 1 mg ml^−1^ (Trujillo et al., 2020). A constant final concentration of 500 μg ml^−1^ of VN was used in our hydrogels. Regarding the relevance of the VN expression levels in NB (Burgos-Panadero, Noguera, et al., 2019; Vicente-Munuera et al., 2020), this system allowed us to regulate the desired amount of VN incorporated, so we can mimic multiple NB ECM conditions. Our cell adhesion studies demonstrated that SK-N-BE (2) adhere and form focal adhesions when seeded on laminin, native VN, and PEGylated VN (Figures 2B,D,E). These results confirm that PEGylation does not reduce VN biological activity, but more importantly, they demonstrate that neuroblastic cells interact with VN to a similar extent than other studied glycoproteins such as laminin, supporting the role of VN in cell migration (Alfano, Franco and Stoppelli, 2022). However, our results do not provide information about focal adhesion signaling activation, so further experiments such as phosphor-FAK focal adhesion protein assessment could deeper evaluate how cells interact with both PEGylated and native VN.

Stiffness is a well-known modulator of tumor behavior and is involved in NB aggressiveness as well as other cancers. However, the limited studies that measured the stiffness of human NB biopsies have reported contradictory results. According to the literature, the stiffness described in soft tissues such as nervous system components range from 0.1 to 1 kPa (Wolf et al., 2013), but human NB biopsies present broader values, from 0.17 to 8.45 kPa (Bao et al., 2022). Critically, the VN/PEG hydrogels reported here recapitulate a small range of physiological stiffness, from 0.42 ± 0.09 kPa to 4.12 ± 2.14 kPa (mean ± SD) (Figures 3A,B), so higher stiffness conditions could be tested in further studies. As previously reported, PEG composition ratios and cell degradation over time regulated hydrogel stiffness (Lutolf and Hubbell, 2003; Almany and Seliktar, 2005; Leslie-Barbick, Moon and West, 2012; Cambria et al., 2015; Jha et al., 2016). Moreover, the stiffness of similar acellular PEG hydrogels has been reported to be stable for up to 9 days (Dobre et al., 2021). In NB, high VN expression correlates with aggressiveness and high stiffness (Burgos-Panadero, Noguera, et al., 2019; Vicente-Munuera et al., 2020), however, VN incorporation did not modify hydrogels stiffness (Figure 3A), as occurs with the incorporation of other full-length proteins (Trujillo et al., 2020; Dobre et al., 2021), this could be due to its lower molecular weight and more globular conformation compared to other ECM proteins. *In vivo*, VN interacts with tumor cells and fibers within the complex TME, which facilitates ECM remodeling and mediates tumor stiffness (Burgos-Panadero, Noguera, et al., 2019). In our models, VN is PEGylated and then covalently bound to a PEG network, so this approximation may not properly recapitulate the VN influence in NB stiffness determination. Nonetheless, our data suggests that VN may be responsible for defining part of the mechanical properties of the hydrogels, simultaneously making them more degradable since the stiffness decreased more over time when VN was present in the hydrogels (Figures 3B,C). In this regard, VN has been reported to be degradable by matrix metalloproteinases such as MMP1, 2, 3, 7 and 9 (Imai, Shikata and Okada, 1995). Besides, VPM is also degraded by MMP1 and 2 (Foster et al., 2017). Therefore, the extra decrease in stiffness for VN/PEG hydrogels may be a combined effect of both VN and VPM degradation by the MMPs synthesized by SK-N-BE (2) (Roomi et al., 2013; Xiang et al., 2015; Mitchell and O’Neill, 2016). Importantly, the stiffness reduction of 10 wt% PEG and VN/PEG hydrogels was 20% higher than 3 wt% PEG and VN/PEG hydrogels, which may indicate that cells reacted to the stiff microenvironment by increasing their ECM degradation activity.

The incorporation of VN as the only bioactive molecule in this system allowed us the study of VN’s specific role within the NB ECM, together with its stiffness. We found that SK-N-BE (2) cells presented the same viability and equivalent cluster growth rate in both stiffness conditions tested (Figures 4A–C), supporting the capability to model the NB cluster development although stiff conditions were more restrictive to cell aggregate allocation (Figure 4D). Thus, the stiff hydrogels provide higher confinement to the cells due to their small pore size which hinders rapid cell growth and migration. Nevertheless, this environment likely promotes metabolic adaptation leading to the increased degradation rate of the stiff hydrogels mentioned above (Figures 3B,C), being that cells may need to promote softer ECM conditions to grow efficiently.

Regarding the evaluation of cell secreted VN diffusion into the culture media, the ELISA kit displayed a VN limit detection of <15,6 ng ml^−1^ and so we were not able to detect VN in some samples (Table 1). We cannot confirm whether negative results were due to a lack in hydrogel number, culture time or VN cell synthesis. In fact, we should consider that cells may adapt their VN production overtime, so they could reduce or even stop their VN synthesis in order to prioritize other biological activities. We have demonstrated that SK-N-BE (2) cells synthesize VN, at least at initial culture times, even when it is already present in the environment (Table 1). More importantly, these results confirm that we can detect VN release in the culture media. In healthy conditions, VN is mainly synthesized by hepatocytes, and it is released into the bloodstream. The mean concentration measured in plasma in healthy subjects has been reported as 200–300 μg ml^−1^ (Clemetson, 1997), and various studies regarding VN concentration in different diseases have been performed (Boyd et al., 1993), so VN could become a liquid biopsy biomarker for high-risk NB. Additionally, confocal image analysis suggests that cells may produce slightly more VN in soft conditions due to less restrictive confinement (Figure 5). However, further experiments other than quantification of fluorescence (integrated density) will be necessary to determine if cells cultured within VN/PEG hydrogels secrete more VN compared to cells within PEG hydrogels. If higher VN integrated density is confirmed in VN/PEG hydrogels, the experiments should assess whether it is due to the presence of both synthesized and PEGylated VN (cells may preferentially grow on PEGylated VN spots or pericellularly reorganize PEGylated VN), or because an increased cell VN synthesis driven by PEGylated VN. Despite no statistical differences, the fact that VN expression is retained *in vitro* conditions indicates that VN/PEG hydrogels are susceptible for translational combinatory therapy studies as occurs with GD2 (KO et al., 2022).

Overall, these data demonstrate that full-length and functional VN can be incorporated into a synthetic hydrogel system that has controlled stiffness and degradation rates, mimicking part of the pathophysiology of high-risk NB tumors. Based on higher cluster and VN integrated densities, together with increased degradation of the stiff hydrogels, we infer that 3 wt% VN/PEG conditions better recreate NB cell behavior. However, cell adaptive response depends on stiffness, time and the genetic background of the cell line, as previously suggested in the literature (López-Carrasco et al., 2020; Monferrer, Sanegre, et al., 2020), so further genetic and drug-testing studies on long-cultured models, using various *MYCN* amplified and/or *ALK* mutated NB cell lines, should be performed to confirm which model better recapitulates high-risk NB behavior. Finally, VN synthesis even in VN rich ECM conditions strengthen the relevance of testing VN targeted preclinical therapies in NB.

## Data Availability

The original contributions presented in the study are included in the article/Supplementary Materials, further inquiries can be directed to the corresponding authors.

## Supplementary material

Supplementary Figure S1 PEGylated VN characterization using a TNBS assay. A. Standard curve of VN (left side) and VN free amines (in the middle) for different PEGylation ratios between VN:MAL and percentage of free amines for different PEGylation ratios shown on the right side.

Supplementary Figure S2 VN distribution and retention after incorporation in the 3D hydrogel. On the top, immunofluorescence images of 3 wt% PEG hydrogels and on the bottom, 10 wt% PEG cryosections. Columns represent PEG, PEG+VN (day 0 and 3) and VN/PEG (day 0 and 3) conditions (scale bar 50 μm). Vitronectin was stained with a Cy3 secondary antibody, shown in red in the images.

Supplementary Figure S3 Insets of FA images (vinculin in red) of SK-N-BE(2) cells seeded on top of unmodified or PEGylated VN for 24h (scale bar: 25 μm).
