# Supplementary figures and images for: Vitronectin-based hydrogels recapitulate neuroblastoma growth conditions
Source: Front Cell Dev Biol. 2022 Oct 11;10:988699. doi: 10.3389/fcell.2022.988699 (PMC9679952; doi:10.3389/fcell.2022.988699)

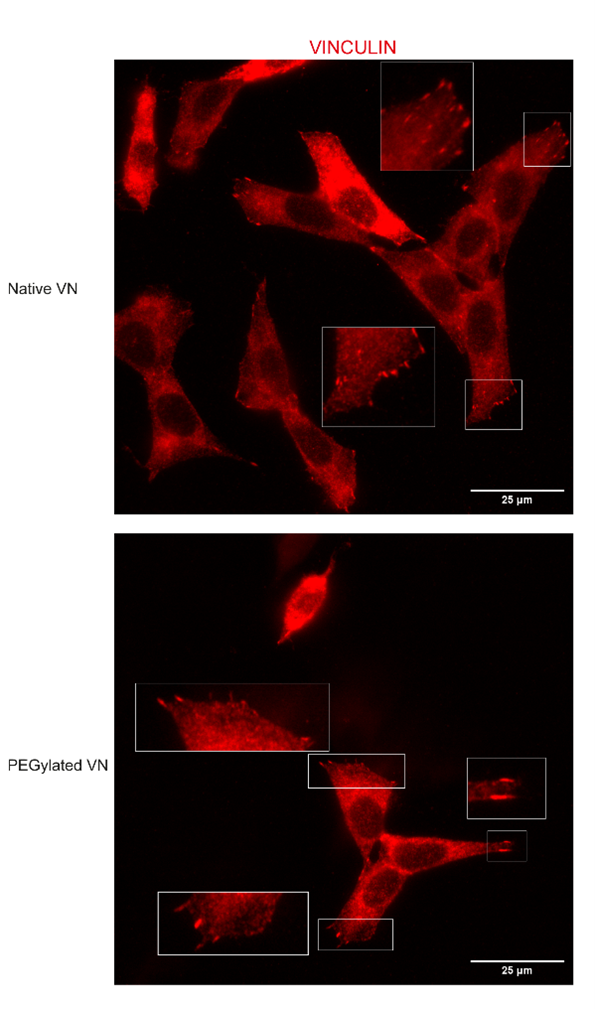

Supplement: Supplementary file 1 [file Image3.TIF]

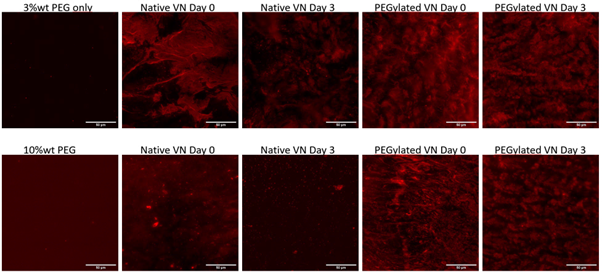

Supplement: Supplementary file 2 [file Image2.TIF]

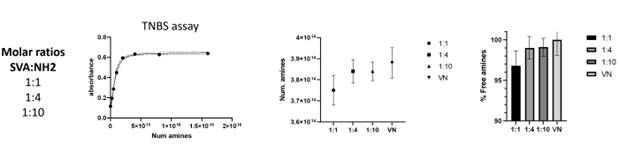

Supplement: Supplementary file 3 [file Image1.TIF]
